# Hyperglycemic microenvironment-driven periodontal tissue defects: pathogenic mechanisms and therapeutic advances

**DOI:** 10.3389/fdmed.2026.1922626

**Published:** 2026-08-04

**Authors:** Shuyu Wang, Wenjun Zhang, Yudong Guo, Zhiyuan Jia, Xiuping Wu, Bing Li, Xuan Jing

**Affiliations:** 1 Shanxi Medical University School and Hospital of Stomatology, Shanxi Province Key Laboratory of Oral Diseases Prevention and New Materials, Taiyuan, China; 2 Shanxi Medical University School and Hospital of Stomatology, Shanxi Province Engineering Research Center of Oral Biomaterials, Taiyuan, China

**Keywords:** diabetes, hyperglycemia, pathogenesis, periodontal therapy, periodontitis

## Abstract

**Background:**

In recent years, the prevalence of diabetes mellitus and periodontal disease has been on the rise, which has drawn scholars' attention to the association between diabetes mellitus and periodontitis.

**Main body:**

Studies have demonstrated that a hyperglycemic microenvironment can notably increase the incidence of periodontitis and the severity of bone loss, impair the regenerative capacity of periodontal tissues, and thereby affect the therapeutic effect of periodontitis. This paper comprehensively reviews the latest research progress regarding the impact of diabetes mellitus on periodontitis and summarizes the current therapeutic strategies for diabetes complicated with periodontitis, including both traditional therapeutic methods and novel therapeutic approaches.

**Conclusion:**

In conclusion, this review provides a systematic overview of the interaction between diabetes and periodontitis as well as related treatments, laying a foundation for subsequent clinical research and practice.

## 1 Introduction

Diabetes mellitus and periodontitis exhibit a close bidirectional interaction: a hyperglycemic microenvironment worsens periodontal tissue damage, while periodontal inflammation further disrupts metabolic balance. This comorbidity has become a major global health issue, as its high incidence and complex pathology place a heavy burden on oral health and overall well-being (1–4). Hyperglycemia drives periodontal tissue destruction by disrupting immune responses, imbalancing bone metabolism, and impairing tissue repair. This forms a vicious cycle that complicates clinical treatment. Traditional therapies, focusing on glycemic control and mechanical debridement, have limitations such as insufficient tissue regeneration, potential drug resistance, and inability to address systemic-local interactions (5, 6). In recent years, innovative treatments integrating precision drug delivery, biological agents, advanced biomaterials, and physical adjuvants have shown great potential to overcome these challenges. This review summarizes the latest research on the pathological link between the two diseases, discusses the drawbacks of traditional therapies, and highlights cutting-edge treatment advances, offering insights into how multi-dimensional strategies can transform the management of this destructive comorbidit (7–9)^.^

### 1.1 Literature search methodology and scope definition

This narrative review searched PubMed, Web of Science, and Scopus for literature published from January 2015 to June 2026. Search keywords included: (“diabetes mellitus” OR “hyperglycemia”) AND (“periodontitis” OR “periodontal disease”) AND (“pathogenesis” OR “immune” OR “osteoclast” OR “osteoblast” OR “stem cells” OR “therapy” OR “biomaterials” OR “regeneration”). Additional articles were identified from reference lists of included studies. Inclusion criteria were: (1) clinical studies; (2) animal models of diabetic periodontitis; (3) *in vitro* studies on periodontal cells under high-glucose conditions; (4) biomaterial and therapeutic strategies. Studies on obesity or metabolic syndrome without confirmed hyperglycemia, and non-periodontal diabetic complications, were excluded. Priority was given to randomized controlled trials, systematic reviews, and high-impact mechanistic studies; preclinical evidence was included when human data were limited. Unless otherwise specified, “diabetes” refers to type 2 diabetes; evidence from type 1 diabetes or animal models is included when it provides unique mechanistic insights and is clearly annotated.

Unlike previous reviews focused on individual mechanisms, this review offers a distinctive contribution in three aspects. First, we integrate local pathogenic pathways (AGEs-RAGE, oxidative stress, NLRP3 inflammasome) with cross-system mechanisms (gut-periodontium axis, inflamm-aging) within a unified framework. Second, we apply graded evidence assessment to distinguish clinically validated strategies from those at preclinical stages, and compare translational bottlenecks across different therapy types. Third, we identify key contradictions in the literature — for instance, while the AGEs-RAGE pathway is well-validated in animal models, targeted interventions have yielded inconsistent clinical benefits; and although stem cells and exosomes show promise, they lack standardized protocols and long-term safety data. Through this framework, this review provides a clear trajectory from mechanistic understanding to clinical translation, rather than a mere compilation of findings.

## 2 Epidemiological characteristics of diabetes mellitus, periodontal disease and their bidirectional association

Latest epidemiological surveys indicate that the global number of people living with diabetes is on a steady rise, with the total number of patients exceeding 537 million currently. It is projected to grow to 783 million by 2045, representing an annual growth rate of approximately 3% to 4%. The International Diabetes Federation's Diabetes Atlas reveals that in 2021, 140.9 million people in China had diabetes, and this figure is expected to increase to 174.4 million by 2045, maintaining China's position as the country with the highest number of people living with diabetes worldwide (1, 10). According to the 2025 data from the World Health Organization (WHO), the number of severe periodontal disease cases exceeds 1 billion, and the prevalence rate among people aged 15 and above is approximately 19%. The prevalence of severe periodontal disease begins in late adolescence, peaks at around the age of 55, and persists into old age. Males and females are affected almost equally. Furthermore, Research has demonstrated that people with type 2 diabetes mellitus have a 2- to 3-fold risk of developing severe periodontal disease compared with non-diabetic individuals (11). Additionally, among people with type 2 diabetes with poorly controlled blood glucose, 68.52% are complicated with severe periodontal disease—a proportion significantly higher than that in people with optimal or well-controlled blood glucose (12). Meanwhile, the implementation of standardized periodontal disease treatment for such people with diabetes can reduce their glycated hemoglobin levels by an average of 0.4% to 0.6% (13). These sets of data fully confirm that there is a significant bidirectional association between diabetes mellitus and periodontal disease.

## 3 The pathogenic mechanisms of diabetes mellitus on periodontal tissue defects

### 3.1 The impact of diabetes Mellitus on immune cell function

The immune system consists of a series of cell populations that perform distinct functions in host defense. Among these cell populations, neutrophils are important participants in the innate immune response; macrophages and monocytes act as core phagocytes; T lymphocytes mediate cellular immunity; and B lymphocytes mediate humoral immunity (14–16).

Neutrophils, acting as the first line of defense in the periodontal immune response, are crucial participants in the innate immune response and possess potent anti-infective capabilities (17). Its antibacterial mechanisms mainly include adhesion, chemotaxis, and phagocytosis, as well as the release of antibacterial substances such as reactive oxygen species (ROS) and lysosomal enzymes, and the formation of extracellular bactericidal networks (18, 19). In the context of diabetes mellitus, neutrophils present obvious functional defects, accompanied by increased release of inflammatory mediators (20, 21). First, their chemotaxis and phagocytosis are impaired: reduced secretion of interleukin-8 hinders neutrophil recruitment and causes accumulation, thereby worsening periodontal damage (22, 23); Second, hyperglycemia enhances protein kinase C activity in neutrophils, which increases ROS and matrix metalloproteinases, induces oxidative stress, and synergistically aggravates periodontal tissue damage (24, 25); Neutrophil apoptosis is delayed (lower caspase-3 activity), and AGEs-RAGE interaction further amplifies the inflammatory response (26); Pro-inflammatory mediators and RANKL/interleukin-17 are upregulated, accelerating alveolar bone resorption and periodontal injury (27–29).

In the context of diabetes mellitus, the physiological activities of macrophages undergo alterations, thereby accelerating the progression of periodontitis and the damage of periodontal tissues. Nuclear factor-*κ*B (NF-*κ*B) pathway activation induces macrophage polarization toward the pro-inflammatory M1 phenotype, promoting pro-inflammatory cytokine (IL-1β, TNF-α) secretion while suppressing anti-inflammatory M2 polarization (30, 31).

This imbalance recruits immune cells, amplifying periodontal inflammation. Specifically,hyperglycemia-induced advanced glycation end products (AGEs) bind to macrophage surface receptors, impairing phagocytic receptor expression and lysosomal enzyme release. This reduces clearance of pathogens like Porphyromonas gingivalis, causing persistent inflammation (32, 33). Moreover,M1 macrophages increase RANKL but decrease osteoprotegerin (OPG) secretion, disrupting the RANKL/OPG balance to accelerate osteoclast activation and alveolar bone resorption (34, 35). Additionally, hyperglycemia stimulates excessive reactive oxygen species (ROS) production in macrophages, triggering oxidative stress. ROS damages periodontal extracellular matrix, activates inflammatory pathway, and inhibits antioxidant enzymes, exacerbating tissue damage (36).

Lymphocytes (including T cells and B cells) are the core components of adaptive immunity. In the context of diabetes mellitus, their functional abnormalities further exacerbate the progression of periodontal lesions. Hyperglycemia and periodontal pathogens synergistically disrupt the balance of T cell subsets: upon activation by antigens and differentiation into effector T cells (Teff cells), T cells initiate a “metabolic reprogramming” process (switching from oxidative phosphorylation to glycolysis) to support the synthesis of effector molecules (37). However, long-term hyperglycemia impairs mitochondrial function, reduces ATP production in CD4+ T cells, disrupts the Treg/Th17 balance, and decreases immune tolerance. Consequently, pro-inflammatory Th1 and Th17 cells become enriched—Th1 cells secrete IFN-*γ* to amplify inflammation, while Th17 cells produce IL-17 to upregulate RANKL expression and accelerate alveolar bone resorption; in contrast, anti-inflammatory Treg cells exhibit diminished capacity to suppress excessive inflammation due to reduced secretion of TGF-*β* and IL-10 (38, 39). B lymphocytes also exhibit impaired function: Hyperglycemia directly disrupts B cell activity, promoting their abnormal proliferation and differentiation into plasma cells as well as the excessive production of pro-inflammatory antibodies. These antibodies activate the complement system and recruit more inflammatory cells to infiltrate the affected site. In addition, the dysfunction of B cells as antigen-presenting cells reduces their antigen-presenting capacity and disrupts the coordination between innate and adaptive immunity (40, 41).

**Figure 1:**
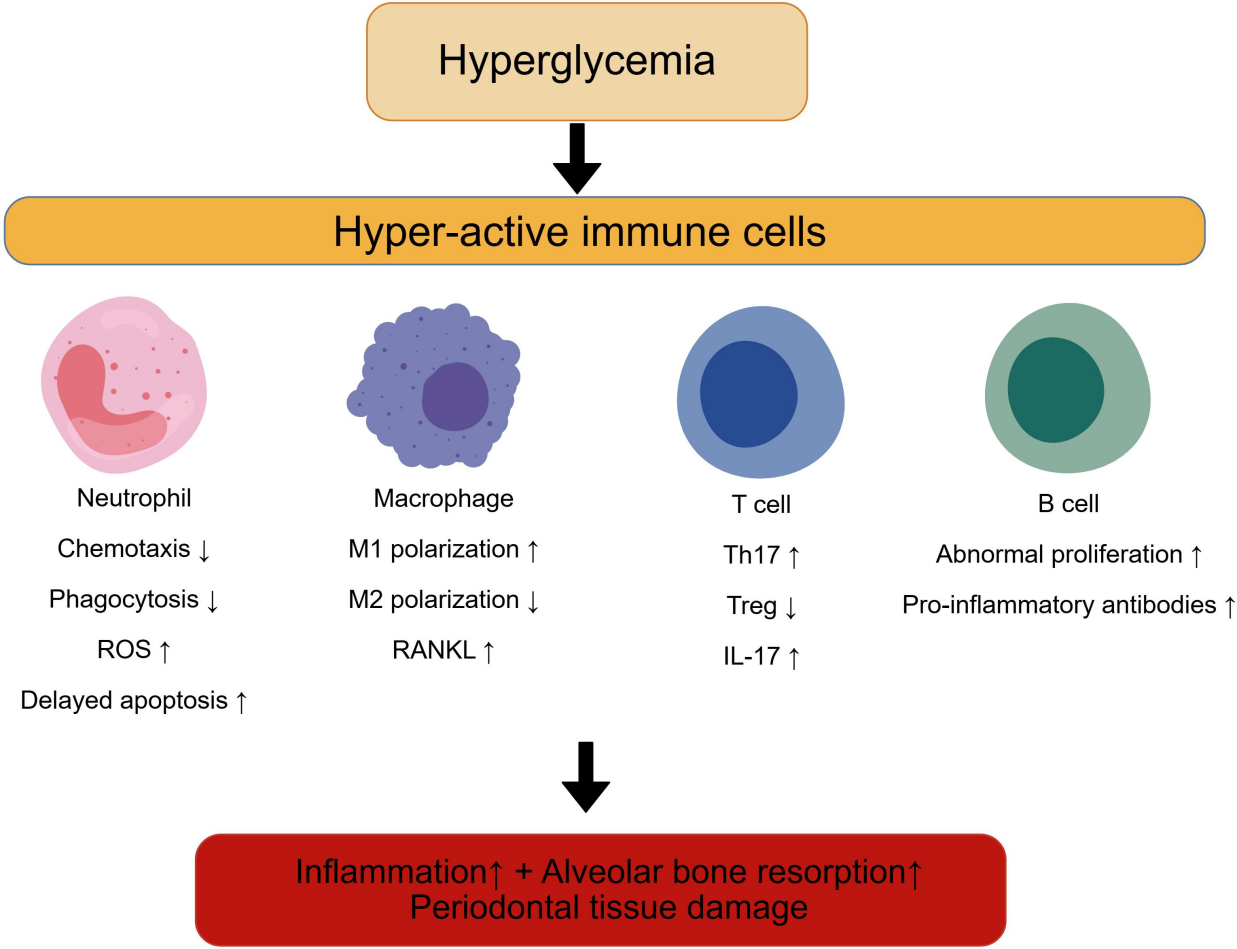
Schematic diagram illustrating immune cell dysfunction in hyperglycemia-associated periodontitis. Hyperglycemia drives functional abnormalities in four major immune cell types: neutrophils (chemotaxis↓, phagocytosis↓, ROS↑, delayed apoptosis↑), macrophages (M1 polarization↑, M2 polarization↓, RANKL↑), T cells (Th17↑, Treg↓, IL-17↑), and B cells (abnormal proliferation↑, pro-inflammatory antibodies↑). These changes collectively promote inflammation↑ and alveolar bone resorption↑, leading to periodontal tissue damage.

However, most of these immune cell findings are derived from animal models or *in vitro* high-glucose cultures; direct evidence from periodontal tissues of diabetic patients remains limited, and the relative contribution of each immune subset to human disease progression is still unclear. These immune cell dysfunction pathways under hyperglycemic conditions are summarized in Figure 1.

### 3.2 The impact of diabetes mellitus on osteoclasts, osteoblasts, and alveolar bone remodeling

Osteoclasts are key mediators of bone remodeling and resorption, playing an essential role in maintaining human bone tissue homeostasis. In the hyperglycemic and inflammatory microenvironment induced by diabetes, osteoclast function is disrupted by imbalanced inflammatory responses and heightened oxidative stress—leading to abnormal activation, enhanced bone-resorbing capacity, accelerated alveolar bone loss, and progressive periodontal tissue damage (42, 43). First, imbalanced inflammatory responses: Diabetes exacerbates periodontal inflammation, resulting in a marked increase in pro-inflammatory factors in the periodontal tissues and body fluids of both type 1 and type 2 diabetic patients. These factors directly stimulate the proliferation and activity of osteoclast precursors while disrupting the RANK-RANKL/OPG pathway balance, which elevates the RANKL/OPG ratio and further promotes osteoclast formation. Experiments have confirmed that in diabetic rats infected with periodontal pathogens, the number of periodontal osteoclasts is 2–4 times higher than that in the control group, accompanied by a more persistent inflammatory response (44–46). Second, metabolic byproduct-related effects: Clinical studies have shown that the expression level of hyperglycemia-derived harmful metabolites is significantly higher in patients with type 1 diabetes complicated by periodontitis than in those with type 2 diabetes, which may indirectly modulate osteoclast activity by amplifying local tissue damage (47, 48). Third, enhanced oxidative stress: The synergistic effect of the hyperglycemic environment and periodontal pathogens induces excessive local production of reactive oxygen species (ROS). Excessive ROS indirectly enhances osteoclast activity by activating pathways such as NF-*κ*B and protein kinase C to promote pro-inflammatory factor secretion. It also acts as a direct signaling molecule, promoting osteoclast activation through the action of inherent NADPH oxidase and exacerbated oxidative stress. Ultimately, this significantly strengthens the bone resorption function of osteoclasts and accelerates alveolar bone destruction (49, 50).

Osteoblasts maintain periodontal health by enabling bone formation and balance, critical for repairing periodontal damage and preventing alveolar bone loss.

Yet diabetes accelerates their apoptosis, disrupting bone homeostasis and worsening bone resorption (51). First, diabetes and periodontal pathogens synergize: pathogen immunity cuts osteoblast numbers, and their apoptosis depletes periodontal ligament, a key osteoprogenitor source. Bacterial stimulation upregulates diabetic osteoblast apoptosis genes by 2-fold, with apoptosis 5x higher than controls—driving impaired new bone formation, reversible via inhibitors (52, 53). Second, elevated TNF-α induces apoptosis: Diabetes increases TNF-α levels, which blocks osteoblast differentiation and directly triggers osteoblast apoptosis via TNFR1 (54, 55). Third, hyperglycemia-induced ROS perturbs Wnt/*β*-catenin and FoxO pathways: excess ROS diverts Wnt/*β*-catenin to FoxO for stress resistance, reducing differentiation-related *β*-catenin and increasing apoptosis. In sum, diabetes inhibits osteoblasts and promotes apoptosis, creating an “accelerated resorption, impaired formation” imbalance with osteoclasts, worsening the periodontitis-hyperglycemia cycle (56, 57).

Furthermore, the dynamic crosstalk between osteoclasts and osteoblasts under diabetic conditions remains incompletely characterized, as most studies provide static snapshots rather than longitudinal observations of bone remodeling dynamics.

### 3.3 The impact of diabetes mellitus on the functions of other periodontal tissue cells

The impairment of periodontal repair function by diabetes is centrally manifested in the targeted damage to two key cell types during the repair process: periodontal ligament stem cells (PDLSCs) and vascular endothelial cells. Dysfunction of these two cell populations leads to nutritional deficiency and regenerative impairment of the periodontal ligament, ultimately resulting in the failure of periodontal tissue repair (58, 59).

As critical repair cells, PDLSCs' proliferation, osteogenic differentiation and damage resistance determine repair efficacy. Diabetes induces PDLSC exhaustion through two core pathways: accelerated death and deviated differentiation. Hyperglycemia triggers excessive ROS production, which has been associated with multiple forms of PDLSC death, including Caspase-3-dependent apoptosis, NLRP3 inflammasome-mediated pyroptosis, and GPX4 downregulation-related ferroptosis. However, it should be noted that the quantitative evidence for these cell death pathways in diabetic periodontitis is primarily derived from *in vitro* high-glucose models and animal studies, and the relative contribution of each pathway to PDLSC dysfunction in human diabetic periodontitis remains to be fully elucidated (60–63). Functionally, hyperglycemia inhibits the Wnt/*β*-catenin pathway, which has been reported to reduce the expression of PDLSC osteogenic markers such as RUNX2 and osteocalcin in experimental models. However, the precise magnitude of this effect varies across studies, and direct evidence from human diabetic periodontal tissues is still limited (64, 65).

Moreover, as the key component of the periodontal vascular network, vascular endothelial cells rely on their proliferation, migration, and angiogenesis-promoting capacities to serve as the core guarantee for oxygen, blood, and nutrient supply required for tissue repair. Hyperglycemia mediates the degradation of hypoxia-inducible factor-1*α* (HIF-1*α*) via reactive oxygen species (ROS), reducing vascular endothelial growth factor (VEGF) expression by 60% and blocking angiogenesis signaling pathways. Dysfunctional endothelial cells release pro-inflammatory factors such as IL-6 and TNF-α, which exacerbate vascular damage and amplify inflammation, forming an “ischemia-inflammation” vicious cycle that ultimately cuts off the nutritional supply essential for periodontal repair (66–68).

Notably, the wide variation in high-glucose concentrations used across *in vitro* studies (ranging from 5 to 50 mM) complicates cross-study comparisons and may contribute to discrepancies in reported PDLSC responses, highlighting the need for standardized experimental conditions.

### 3.4 Cross-system regulatory mechanisms: the gut-periodontium axis and inflamm-aging

Beyond the local immune and bone metabolic disturbances discussed above, emerging evidence indicates that gut microbiota dysbiosis contributes to the systemic inflammatory amplification in diabetic periodontitis through the “gut-periodontium axis.” Hype (69) rglycemia increases intestinal permeability, facilitating the translocation of lipopolysaccharides into the circulation, which activates Toll-like receptor signaling and further exacerbates the inflammatory burden at remote periodontal sites (70). Concurrently, persistent hyperglycemia drives an “inflamm-aging” phenotype, characterized by chronically elevated pro-inflammatory cytokines (IL-6, TNF-α) and progressive immune cell exhaustion (71). These broader mechanisms provide the theoretical foundation for the microbiome-targeted and systemic anti-inflammatory strategies discussed in the therapeutic sections below.

## 4 The therapeutic advances of diabetes mellitus on periodontal tissue defects

### 4.1 Traditional therapeutic approaches: periodontal treatment based on glycemic control

#### 4.1.1 Pharmacological treatment (balancing systemic glycemic control and local periodontal improvement)

Pharmacological treatment for diabetic periodontitis targets balanced systemic glycemic control and local periodontal improvement, focusing on blood glucose stabilization and periodontal pathology alleviation. Systemic glycemic stability relies mainly on hypoglycemic agents, with two core local drugs: insulin precisely regulates blood glucose, mitigates hyperglycemia-induced oxidative stress and pro-inflammatory cytokine release, reduces periodontal pathogen load and pathogenicity (72, 73). Modulates the PI3 K/Akt pathway to improve osteogenic differentiation of hyperglycemic periodontal ligament stem cells (PDLSCs), and promotes tissue repair; locally administered biguanides regulate blood glucose, inhibit periodontal tissue degradation and boost osteogenesis (74). Adjunctive therapies include antibacterial agents like antimicrobial peptides and novel materials (nano-silver, zinc oxide nanoparticles, zinc-based zeolitic imidazolate frameworks) (75, 76). Anti-inflammatory and immunomodulatory agents including non-steroidal anti-inflammatory drugs, natural compounds and dexamethasone that exert direct anti-inflammatory effects, as well as interleukin-1 receptor antagonist-loaded hydrogels and granulocyte-macrophage colony-stimulating factor that regulate immune function and optimize the periodontal microenvironment (77, 78). and antioxidant stress agents such as natural polyphenols like curcumin, which alleviate oxidative damage through inhibiting the NF-*κ*B signaling pathway and activating the Nrf2 signaling pathway to create favorable conditions for periodontal tissue regeneration (79, 80). Beyond medications, lifestyle interventions are crucial adjuncts for blood glucose control, synergizing with drugs to support periodontal repair. These include low-glycemic-index diets (whole grains, legumes, leafy greens) with limited refined sugars and processed foods, 150 min of weekly moderate aerobic exercise (brisk walking, cycling) to enhance insulin sensitivity (patients with active periodontal inflammation may be advised to avoid high-impact exercise that could traumatize gingival tissues, although direct evidence supporting this precaution in diabetic periodontitis patients remains limited.), healthy weight maintenance, 7–8 h of daily sleep, and smoking cessation and alcohol restriction to reduce metabolic impacts on blood glucose (81, 82).

#### 4.1.2 Local periodontal therapy (mechanical debridement and adjunctive interventions)

On the basis of effective blood glucose control, periodontal therapy should adhere to the core strategy of “mechanical debridement plus adjuvant antibacterial therapy” and optimize the procedures in light of the characteristics of slow periodontal healing and susceptibility to bleeding in diabetic patients. Ultrasonic supragingival scaling combined with scaling and root planing (SRP) is the core method of basic therapy, and its efficacy is closely correlated with the level of blood glucose stability. Randomized controlled trials have confirmed that SRP can significantly improve periodontal probing depth (PPD) and attachment loss (AL) in diabetic patients, reduce serum inflammatory factors, and also bring about an average 0.4% decrease in glycated hemoglobin (83). After 12 months of treatment, patients with a baseline glycated hemoglobin level > 8% achieved more remarkable glycemic control effects, and the detection rate of pathogenic bacteria was also significantly reduced, which verifies the synergistic benefits of the “glycemic control-periodontal therapy” regimen (84). Clinical procedures should be performed gently to minimize trauma, and the follow-up interval should be extended from 4 to 6 weeks to 8 weeks to facilitate the monitoring of healing progress. For adjuvant therapy, antibacterial photodynamic therapy (aPDT) utilizes a 670 nm laser to activate photosensitizers for eradicating drug-resistant bacteria, and its efficacy is superior to that of doxycycline. Locally, 2% chlorhexidine gargle can be used for a short duration (≤ 2 weeks), and alternating its application with metronidazole gel can enhance antibacterial efficacy and reduce the risk of drug resistance (85–87).

Traditional treatment for diabetic periodontitis has obvious limitations: its efficacy depends heavily on blood glucose control, with poor results and high recurrence in patients with unstable blood glucose, and drug efficacy varies greatly among individuals due to metabolic differences; it lacks means to actively promote periodontal regeneration, failing to reverse alveolar bone resorption, and healing is mostly fibrous (88); adjuvant antibacterial therapies easily cause flora imbalance or drug resistance, while physical methods cannot completely eliminate deep pathogenic bacteria; the long treatment cycle and frequent follow-ups require high patient compliance, and those with complications tend to discontinue treatment; clinically, gentle operation (for patients prone to bleeding) conflicts with thorough debridement, making it hard to balance safety and efficacy, thus requiring optimization with new technologies (89).

A critical caveat is that the efficacy of conventional therapy heavily depends on patient compliance with glycemic control, and recurrence rates are likely underestimated in real-world settings due to the lack of long-term follow-up data in most published studies.

### 4.2 Innovative therapeutic approaches

Traditional subgingival scaling and root planing (SRP) have limited efficacy due to the difficulty in breaking through the pathological barrier induced by hyperglycemia. In recent years, novel therapies centered on “targeting pathological microenvironment + local-systemic synergistic regulation” have covered multiple fields and shown promising preclinical and early clinical potential, although large-scale randomized controlled trials are still needed to confirm their efficacy in routine practice.

#### 4.2.1 Novel biologic therapy

The glucose-responsive transformable complex can target the hyperglycemic microenvironment, eliminate periodontal biofilms and alleviate inflamm-aging, thus providing a novel therapeutic regimen with both targeting ability and effectiveness for diabetic periodontitis (90)，targeting the inflamm-aging pathway as discussed in Section 3.4. The traditional Chinese medicine compound Kouqiangjie Formula can regulate inflammation and inhibit osteoclast differentiation through multiple targets, ameliorate periodontal tissue damage in a hyperglycemic microenvironment, and thus serves as a promising exploration for the novel pharmacological treatment of diabetic periodontitis (91). Umbilical cord mesenchymal stem cells (UCMSCs) can promote bone regeneration in diabetic rats with periapical periodontitis by upregulating the expression of Runx2 and Osterix, thus providing a reference for the cell therapy of diabetic periodontitis (92). Thioredoxin-1 (Trx1) can promote the osteogenic differentiation of periodontal ligament stem cells, reduce reactive oxygen species (ROS) production, and significantly repair alveolar bone defects in mice with diabetic periodontitis by activating the Wnt/*β*-catenin signaling pathway (93). Extracellular vesicles are key mediators linking periodontal disease and diabetic complications, and can regulate the inflammatory and immune processes of diabetic periodontitis. Through functional modification, extracellular vesicles can be developed into cell-free therapeutic agents targeting the periodontal microenvironment, providing a novel therapeutic direction for diabetic periodontitis (94). The combined delivery of periodontal ligament stem cells (PDLSCs) and metformin mediated by organic cation transporters (OCTs) can target and regulate the osteogenic differentiation and anti-inflammatory capacity of stem cells in a hyperglycemic microenvironment (95).

Schwann cell-derived exosomes functionalized with natural polyphenols can serve as a temporal neuromodulation strategy for diabetic periodontitis therapy. These modified exosomes are capable of targeting and regulating the neuro-immune-osteogenic network of periodontal tissues, and ameliorating periodontal tissue damage in a hyperglycemic microenvironment through the synergistic effects of antibacterial activity, anti-inflammation and pro-tissue regeneration (96). Garlic-derived exosome-like nanovesicles (GaELNs) alleviate inflammatory responses and alveolar bone resorption in diabetic periodontitis by activating the PHGDH/PI3 K/AKT pathway, improving mitochondrial function, and regulating metabolic reprogramming (97).

In addition, targeted gene therapy and gene editing have emerged as cutting-edge biotherapeutic strategies for diabetic periodontitis by modulating key pathogenic genes involved in inflammation and osteogenic differentiation. The CRISPR-Cas9 system can knockout hyperactivated inflammatory genes in periodontal ligament stem cells (PDLSCs), thereby enhancing the osteogenic potential of these cells under hyperglycemic conditions (98). Small interfering RNAs (siRNAs) that silence RANKL—a core gene driving osteoclast differentiation—have also been shown to significantly inhibit alveolar bone resorption in diabetic periodontitis models (99).

Novel biological agents offer an innovative therapeutic approach for diabetes-associated periodontitis: multi-active phlorotannins can enhance the activity of antimicrobial peptide LL-37, achieving both antibacterial effects and periodontal tissue regeneration in diabetic conditions; agents targeting the NLRP3 inflammasome reduce alveolar bone loss by inhibiting osteoclast differentiation. With strong targeting and high biocompatibility, these agents make up for the deficiencies of traditional therapies in tissue regeneration and precise anti-inflammation, serving as key carriers linking antibacterial, anti-inflammatory and osteogenic effects (100, 101).

In addition, gut microbiota modulation has emerged as a promising systemic biotherapeutic strategy for diabetic periodontitis by targeting the gut-periodontium axis. Oral supplementation with probiotics remodels the dysbiotic gut microbiome, strengthens intestinal barrier function, and reduces the translocation of pathogenic bacteria and circulating pro-inflammatory cytokines (102)，modulating the gut-periodontium axis as introduced in Section 3.4.

A randomized controlled trial demonstrated that combining probiotics with initial periodontal therapy significantly reduces periodontal pathogens and improves clinical indices in diabetic patients. Fecal microbiota transplantation (FMT) has also been validated in animal models to reverse gut-periodontium axis imbalance, alleviate oxidative stress, and restore alveolar bone volume in diabetic periodontitis, offering a novel option for refractory cases (103).

#### 4.2.2 Novel biomaterial therapy

**Figure 2:**
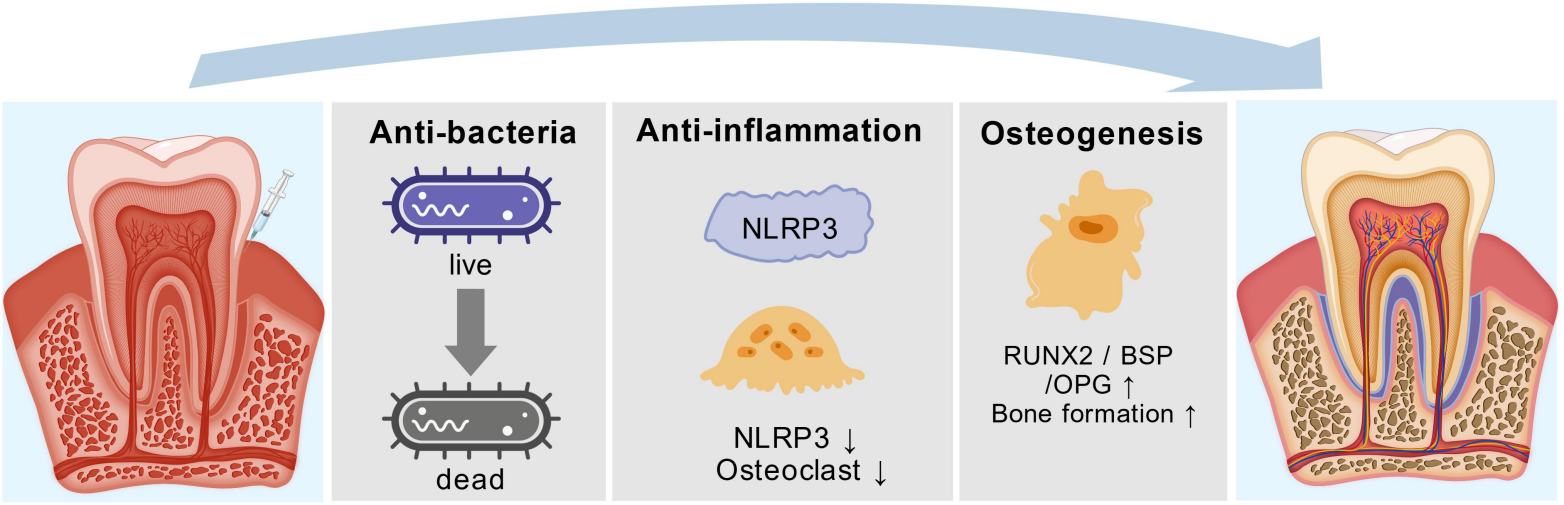
Multi-effect regulation of periodontal lesions via local injection of bioactive agents. Schematic illustrating the three core therapeutic functions of bioactive agents in diabetic periodontitis: antibacterial (LL-37↑), anti-inflammatory (NLRP3↓/osteoclast↓), and pro-osteogenic (RUNX2/BSP/OPG↑/bone formation↑) effects. Figure created by the authors.

As core carriers for precise delivery, hydrogel-based materials play a crucial role in the treatment of diabetic periodontitis due to their excellent biocompatibility and drug-loading capacity. The CHX/EGCG nanoparticle composite hydrogel is a typical representative: it self-assembles chlorhexidine acetate (CHX), a broad-spectrum antibacterial agent, and epigallocatechin gallate (EGCG), a potent antioxidant, into nanoparticles via hydrogen bonding, which are then encapsulated in an injectable and self-healing hydrogel, effectively enhancing drug stability and biocompatibility. During treatment, CHX can disrupt the cell membranes of pathogenic bacteria such as Porphyromonas gingivalis to achieve broad-spectrum antibacterial effects; EGCG can scavenge over 80% of reactive oxygen species (ROS) to protect periodontal cells; and the hydrogel can precisely fill periodontal pockets and realize sustained drug release. The GOE1 immunomodulatory hydrogel, which has a similar mechanism of action, also encapsulates CHX/EGCG self-assembled nanoparticles. It integrates antibacterial, antioxidant, and anti-inflammatory properties, and can significantly alleviate tissue inflammation and alveolar bone loss in diabetic periodontitis (7). Zn-V-Si-Ca glass nanoparticle hydrogel microneedles combine zinc and vanadium ion-doped bioactive glass with antioxidant components to construct a biodegradable microneedle array. Its greatest advantage is the ability to directly penetrate the gingival mucosal barrier and precisely deliver therapeutic components to the deep part of periodontal pockets, solving the problem of poor permeability in traditional drug administration. This microneedle system has the combined effects of antibacterial, anti-inflammatory and osteogenic promotion, as shown in Figure 2.

Other functionalized hydrogels have further expanded the therapeutic dimensions: Resveratrol-loaded solid lipid nanoparticle-enhanced hyaluronic acid hydrogel (RSV@CL Gel) achieves therapeutic effects through multi-targeted actions including anti-inflammation, antioxidant activity, and osteogenesis promotion; ZnO nanoparticle-doped chitosan hydrogel can effectively inhibit the growth of Porphyromonas gingivalis and simultaneously upregulate the expression of osteogenic markers such as Runx2 and OSX, realizing the synergy between antibacterial activity and osteogenesis promotion in the diabetic microenvironment (104, 105); the GelMA-BC-PL hybrid hydrogel bioadhesive, which contains aldehyde-functionalized bacterial cellulose short nanofibers and *ε*-polylysine, achieves periodontal tissue adhesion via Schiff base reaction and integrates the functions of antibacterial activity, tissue adhesion, and tissue regeneration; the polyphenol-mediated redox-active alginate/gelatin hydrogel innovatively integrates a conductive poly(3,4-ethylenedioxythiophene)-polydopamine silk microfiber network and a sustained hydrogen sulfide (H₂S) release system, realizing the synergistic effect of H₂S gas and bioelectricity; after loading liraglutide, the poly(lactic-co-glycolic acid)/hyaluronic acid (PLGA/HA) sustained-release hydrogel can target and inhibit the necroptosis pathway in diabetic periodontitis (106, 107). In addition, as an adjuvant to scaling and root planing (SRP), hyaluronic acid gel is used for the treatment of stage II periodontitis in patients with type 2 diabetes. It can significantly improve periodontal indicators such as clinical attachment level and probing depth, reduce glycated hemoglobin levels, and enhance the efficacy of non-surgical periodontal therapy (108).

Patch-based materials, characterized by long-acting colonization and targeted release, are suitable for the local sustained treatment of diabetic periodontitis. The Met@TA-ZIF-PSG composite patch loads metformin via a tannic acid-modified carrier and combines with a silk fibroin/gelatin matrix to achieve long-acting drug release. It can not only promote the polarization of macrophages to the reparative M2 phenotype and protect periodontal ligament stem cells from hyperglycemia-induced damage but also further enhance osteogenic differentiation through the released zinc ions. The acerola-mediated silver nanoparticle gel focuses on potent antibacterial activity; it exhibits stable antibacterial effects against pathogenic bacteria associated with periodontitis in both diabetic and non-diabetic patients. With lower minimum inhibitory concentration (MIC) and minimum bactericidal concentration (MBC), it has better antibacterial efficacy than copper oxide nanoparticle gel, providing a new option for the precise antibacterial treatment of periodontitis (109).

**Figure 3:**
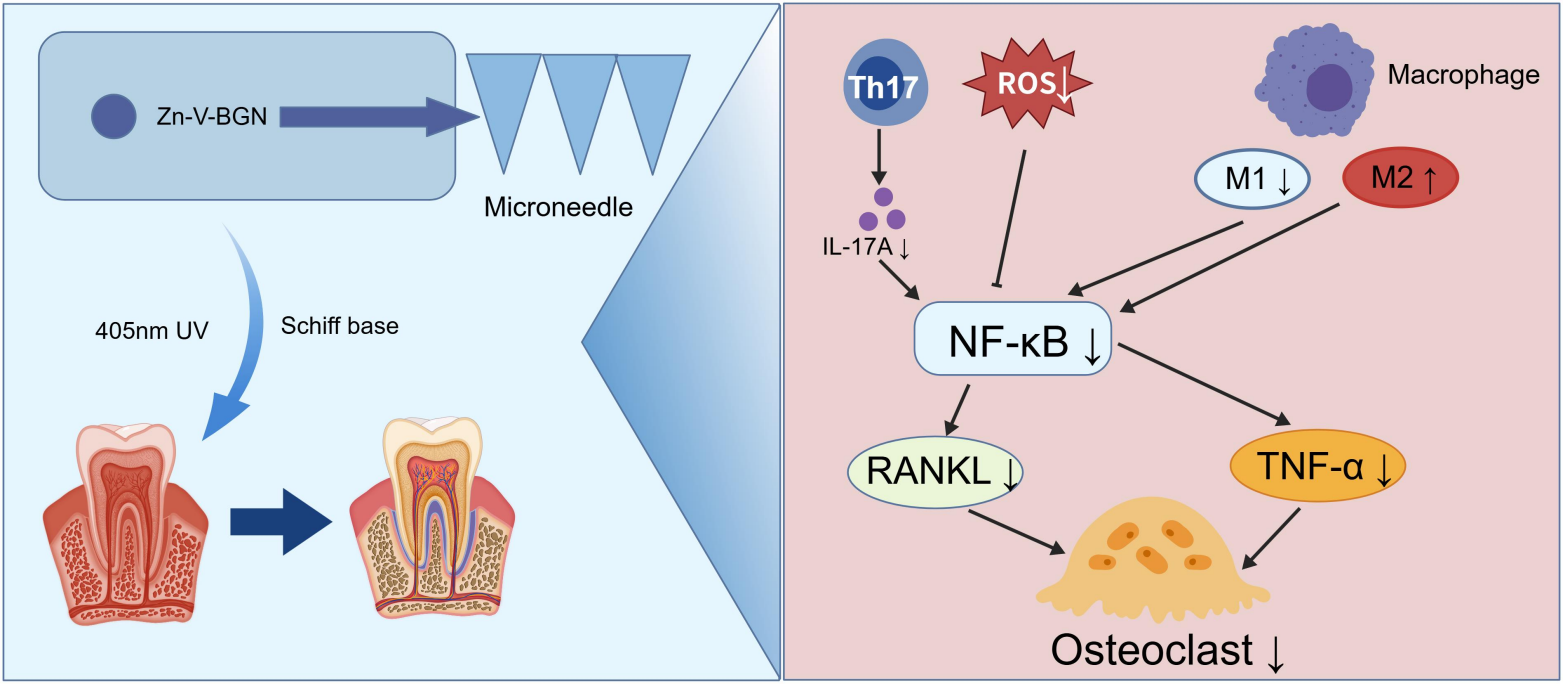
Zn-V-BGN hydrogel microneedles modulate inflammation and bone metabolism in diabetic periodontitis. The Zn-V-BGN microneedle system penetrates the gingival mucosal barrier to deliver therapeutic components. The left panel shows the microneedle components (Zn-V-BGN, microneedle, 405 nm UV, Schiff base). The middle panel depicts the Th17 pathway (Th17 → ROS↓ → IL-17A↓ → NF-*κ*B↓ → RANKL↓ → osteoclast↓), and the right panel depicts the macrophage pathway (macrophage → M1↓/M2↑ → TNF-α↓ → osteoclast↓). Figure created by the authors.

Microneedle-based materials address the issue of poor permeability of traditional drugs by penetrating the gingival mucosal barrier, enabling precise drug delivery to deep periodontal tissues. Polylactic acid (PLA)-based microneedles reinforced with tunicate cellulose nanocrystals (TCNCs) (TCNC-g-PLA@PLA-MNs) are functionalized with polydopamine to load irisin and interleukin-1 receptor antagonist, thereby improving diabetic periodontitis through the dual effects of anti-inflammation and osteogenesis promotion (110). The bilayer microneedle (d-MN@MOF) patch integrates magnesium-based metal-organic frameworks (Mg-MOFs), glucose oxidase (GOX), and a methacrylated gelatin (GelMA)/nano-hydroxyapatite (nHA) substrate. It can not only penetrate the gingiva and reach deep into the periodontal pockets but also reduce local blood glucose levels via GOX (111); meanwhile, Mg-MOFs scavenge reactive oxygen species (ROS) and release magnesium ions to promote angiogenesis, ultimately achieving the synergistic regeneration of periodontal soft and hard tissues. The immune and bone metabolism regulatory network mediated by Zn-V-BGN microneedles is illustrated in Figure 3.

#### 4.2.3 Novel physical therapy

Diode laser-assisted scaling and root planing (SRP) has demonstrated significant value in the treatment of diabetic periodontitis. A systematic review and meta-analysis confirmed that this therapy can effectively improve probing depth (PD), clinical attachment level (CAL) in patients, and exert a positive effect on the control of glycated hemoglobin (HbA1c), providing strong evidence for the clinical application of lasers (112). Another prospective randomized controlled trial also indicated that diode laser, as a novel therapeutic approach for type 2 diabetes mellitus (T2DM) patients with chronic periodontitis, can optimize periodontal clinical indicators and assist in stabilizing blood glucose, showing promising clinical prospects (113). Additionally, diode lasers with a wavelength range of 800–980 nm, when used as an adjuvant to SRP, can also enhance the therapeutic effect on chronic periodontitis, and are particularly significant for periodontal tissue repair in patients with concurrent diabetes (114).

The innovative application of laser therapy is also reflected in the development of specific wavelengths: 445 nm blue light laser combined with SRP can more significantly reduce the number of pathogenic bacteria such as Porphyromonas gingivalis, improve periodontal indicators, and is applicable to patients with diabetic periodontitis (115). The efficacy of laser-assisted open flap debridement (OFD) in periodontitis treatment has also been verified; subgroup analysis of patients with diabetic periodontitis showed that this therapy can improve the effect of surgical treatment, providing new ideas for clinical practice (116).

Beyond high-intensity laser debridement, photobiomodulation therapy (PBM) — a non-thermal low-level laser intervention — is an effective adjuvant for diabetic periodontitis (117). Wavelengths like 660 nm and 808 nm reduce periodontal ROS and pro-inflammatory cytokines (IL-6, IL-12p70), while enhancing anti-inflammatory IL-10. In hyperglycemic rats, PBM lessens alveolar bone loss and improves bone density, with 660 nm laser optimal for reducing linear bone loss (118). It also boosts high-glucose-resistant periodontal stem cell viability and osteogenic markers. Combined with SRP, PBM improves diabetic patients' PD and CAL better than SRP alone (119).

Beyond laser therapy, other physical treatment methods also have their unique advantages: the combined application of ultrasonic scalers and minocycline hydrochloride can more effectively improve periodontal conditions, reduce the level of inflammatory factors, and ensure higher safety in patients with diabetic periodontitis compared with single ultrasonic scaling. Antimicrobial photodynamic therapy (PDT) as an adjuvant to subgingival scaling and root planing can improve the probing depth of T2DM patients with periodontitis in the short term, but its effects on clinical attachment level, bleeding on probing, and glycated hemoglobin are not significant, which provides references for defining the clinical application boundary of this therapy.

Non-surgical periodontal basic treatment itself contributes positively to blood glucose control in T2DM patients with periodontitis, and the effect is more obvious in patients with higher baseline glycated hemoglobin levels; when combined with zinc and magnesium supplementation, it can further regulate oxidative stress levels and enhance the activity of antioxidant enzymes. Even physical exercise has shown potential value: in a diabetic rat model induced by high-fat diet/streptozotocin (HFD/STZ), aerobic exercise can improve blood glucose and inflammatory status, and effectively delay the progression of periodontitis (120)^.^ A study on T2DM patients with stage II periodontitis found that non-surgical periodontal treatment with hyaluronic acid gel is superior to traditional treatment without hyaluronic acid gel in improving periodontal conditions (121). In addition, high-intensity diode laser-assisted non-surgical treatment can long-term improve the clinical indicators of patients with diabetic periodontitis and inhibit the growth of periodontal pathogenic bacteria (122); the long-term effect of diode laser-assisted treatment on T2DM patients with aggressive periodontitis has also been confirmed, which can significantly delay periodontal tissue destruction. Although non-surgical periodontal treatment can improve the glycated hemoglobin level of T2DM patients with moderate to severe periodontitis, its improvement effect on systemic indicators such as high-sensitivity C-reactive protein and oxidative stress markers does not reach statistical significance (122).

#### 4.2.4 Novel systemic therapy

The new systematic treatment methods for diabetes are currently advancing toward automation, precision, and mechanism targeting, forming a diverse treatment system centered on technological innovation and drug research and development. In the field of insulin delivery, automated closed-loop insulin delivery systems have become a major breakthrough in the treatment of type 1 diabetes. From early hybrid closed-loop systems to the new generation of fully automated systems, these systems significantly enhance the stability and safety of blood glucose control by real-time blood glucose monitoring and dynamic adjustment of insulin infusion (123). Relevant network meta-analyses have confirmed the efficacy differences among different types of hybrid closed-loop systems in patients with type 1 diabetes, providing evidence-based basis for individualized clinical selection (124). Moreover, this technology also demonstrates prominent value in blood glucose control for the management of type 1 diabetes during pregnancy, offering a new approach for the treatment of special populations (125). For patients with type 2 diabetes, automated insulin delivery systems also show significant advantages. Multicenter randomized trials have confirmed that these systems can effectively reduce the level of glycated hemoglobin, increase the proportion of time when blood glucose reaches the target, and break the application limitations of traditional insulin therapy in adult patients with type 2 diabetes (126).

**Figure 4:**
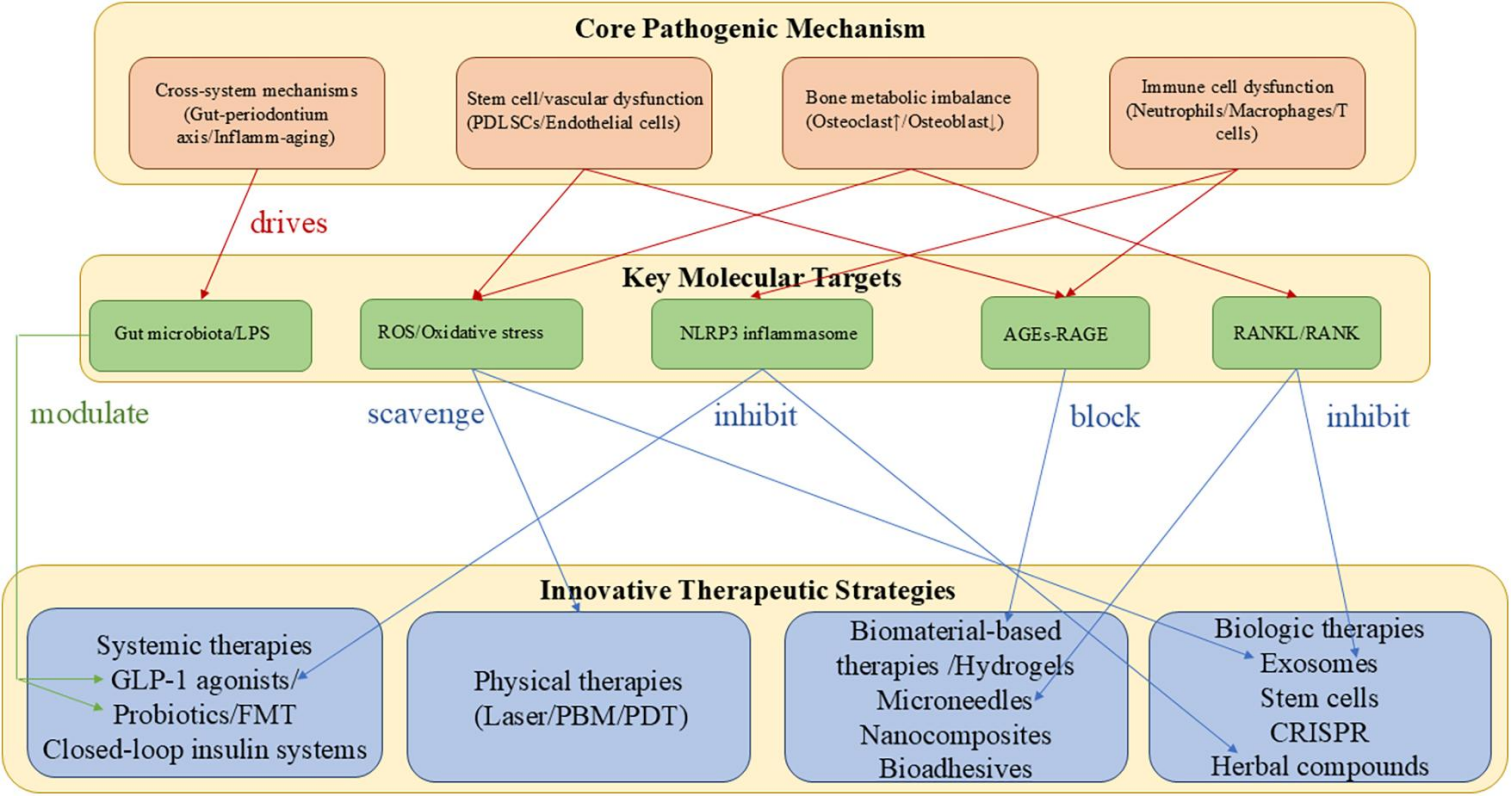
Integrated framework of innovative therapeutic strategies for diabetic periodontitis: from pathogenic mechanisms to molecular targets and therapeutic interventions. The lower tier summarizes the core pathogenic mechanisms, including immune cell dysfunction, osteoclast-osteoblast imbalance, stem cell/endothelial dysfunction, and cross-system mechanisms (gut-periodontium axis and inflamm-aging). The middle tier indicates key molecular nodes targeted by current and emerging therapies, including AGEs-RAGE, NLRP3 inflammasome, ROS/oxidative stress, RANKL/RANK, and gut microbiota/LPS. The upper tier classifies therapeutic modalities into biologic therapies (exosomes, stem cells, CRISPR, herbal compounds), biomaterial-based strategies (hydrogels, microneedles, nanocomposites, bioadhesives), physical adjuvants (laser, photobiomodulation, photodynamic therapy), and systemic interventions (GLP-1 receptor agonists, probiotics/fecal microbiota transplantation, closed-loop insulin systems). Arrows denote mechanistic links from pathological drivers to targetable nodes and therapeutic interventions. Figure created by the authors.

The research and application of GLP-1-based therapies have revolutionized the treatment landscape of diabetes. Drugs represented by semaglutide and tirzepatide, relying on clear mechanisms of action, not only lower blood glucose but also bring additional benefits such as weight loss. Relevant studies have not only in-depth explored their dose-dependent efficacy but also proposed the potential of GLP-1 receptor agonists as an alternative option for selected patients, although metformin remains the current standard of first-line therapy according to most major guidelines. These systemic effects, including anti-inflammatory actions beyond glycemic control, are consistent with the cross-system mechanisms discussed in Section 3.4. The research and development of the next generation of GLP-1 drugs are further expanding toward the collaborative treatment of diabetes and associated diseases such as obesity. In terms of etiological treatment for type 1 diabetes, immunotherapy based on autoimmune mechanisms has achieved key progress. Teplizumab, as a representative drug, effectively preserves the *β*-cell function of patients by precisely regulating immune responses, delays disease progression, and lays a foundation for the realization of “disease-modifying therapy” for diabetes (127). The in-depth analysis of the autoimmune mechanisms of type 1 diabetes also provides a solid theoretical support for the subsequent research and development of immunotherapeutic drugs (128). In addition, new hypoglycemic drugs such as imeglimin provide a new alternative to traditional insulin therapy for patients with type 2 diabetes by targeting the enhancement of glucose-dependent insulin secretion and improving *β*-cell function. The consensus recommendations on the clinical application of various new treatment technologies further standardize the treatment path and promote the transformation of these innovative methods from research to widespread clinical practice (129). A summary of the classification, associations, and core strategies of all the aforementioned innovative therapeutic approaches is visually presented in Figure 4.

Despite the considerable enthusiasm surrounding these emerging strategies, only a few have progressed to early-phase clinical trials, and their long-term safety, cost-effectiveness, and feasibility in routine periodontal practice remain largely unknown. Rigorous head-to-head comparisons with conventional therapy are urgently needed before any recommendation for clinical adoption can be made.

Beyond glycemic control, GLP-1 receptor agonists (GLP-1RAs) exert direct anti-inflammatory effects on periodontal tissues. Preclinical studies have shown that GLP-1RAs suppress NF-*κ*B activation and reduce IL-6, TNF-α, and IL-1β production in periodontal cells independent of glucose-lowering actions (130). Clinical data reveal that diabetic patients on GLP-1RA therapy have lower periodontal probing depths and less bleeding on probing compared with those on other hypoglycemic agents, even after adjusting for HbA1c levels. Mechanistically, GLP-1RAs promote M2 macrophage polarization, inhibit RANKL-induced osteoclast differentiation, and enhance PDLSC osteogenesis via the cAMP/PKA/CREB pathway (131). These pleiotropic effects position GLP-1RAs as promising dual-purpose agents for diabetic periodontitis, although prospective RCTs with periodontal primary endpoints are needed (132).

### 4.3 Evidence-level comparison and translational bottleneck analysis

To objectively assess the translational readiness of various therapeutic strategies, this review classifies the evidence into three tiers: established clinical evidence (large RCTs), early-stage clinical evidence (small-sample clinical studies), and preclinical evidence (animal or *in vitro* studies). The model systems, outcomes, safety concerns, and clinical readiness of each strategy are summarized in Table 1. This framework helps identify therapies near clinical translation and which require further validation.

**Table 1:** Overview of therapeutic interventions for diabetic periodontitis.

Intervention	Model/Study type	Key outcomes	Main safety concerns	Evidence level	Clinical readiness	Reference(s)
SRP + glycemic control	Multiple RCTs (*n* > 500)	PPD ↓, CAL ↑, HbA1c ↓ ∼0.4%	Low	Established clinical	Already in clinical use	(86, 87)
Diode laser + SRP	Small-sample RCTs (*n* = 40-80)	PPD ↓, CAL ↑, HbA1c ↓	Low-moderate	Early-stage clinical	Partial clinical use	(118, 119)
Photobiomodulation (PBM)	*In vitro*/Animal/Small RCT	PDLSC differentiation ↑ (*in vitro*); Bone loss ↓ (animal); PD, CAL improved (Meta-analysis)	Low	Early-stage clinical	Exploratory	(123–125)
CHX/EGCG composite hydrogel	Diabetic rat models	Antibacterial, ROS scavenging >80%, bone loss ↓	Moderate (degradation)	Preclinical	Preclinical stage	(7)
Zn-V-BGN microneedles	Diabetic rat models	Antibacterial, anti-inflammatory, osteogenic	Moderate (local irritation)	Preclinical	Preclinical stage	(109)
RSV@CL hydrogel	Diabetic rat models	Anti-inflammatory, antioxidant, osteogenic	Moderate (degradation)	Preclinical	Preclinical stage	(110)
Stem cells (UCMSCs)	Diabetic rat models	Runx2 ↑, Osterix ↑, bone regeneration ↑	High (tumorigenicity)	Preclinical	Preclinical stage	(96)
Exosomes (GaELNs)	Diabetic rat models	Anti-inflammatory, anti-resorptive	Moderate (immunogenicity)	Preclinical	Preclinical stage	(101)
CRISPR-Cas9 gene editing	*In vitro*/animal models	Inflammatory gene knockout, osteogenesis ↑	High (off-target effects)	Preclinical	Preclinical stage	(102, 104)
Probiotics/FMT	Small RCTs/animal models	Periodontal pathogens ↓, clinical indices ↑	Low-moderate	Early-stage clinical	Exploratory	(107, 108)
GLP-1 receptor agonists	Large T2DM RCTs with periodontal subgroups	HbA1c ↓, periodontal parameters improved	Low-moderate	Established (glycemic)/Early (periodontal)	Approved for T2DM; periodontal indication pending	(37, 38, 133)

#### 4.3.1 Evidence level definitions

##### 4.3.1.1 Established clinical evidence

Supported by ≥2 large-scale RCTs (≥100 participants per arm) or systematic reviews/meta-analyses (≥5 RCTs included), with statistically significant and clinically meaningful outcomes (*P* < 0.05).

Early-stage clinical evidence: Supported by small-sample RCTs (<100 participants per arm), prospective cohort studies (≥30 participants), or case series (≥10 cases).

##### 4.3.1.2 Preclinical evidence

Supported by animal models (e.g., db/db mice, STZ-induced diabetic rats) or *in vitro* studies (e.g., high-glucose cultured PDLSCs, macrophages), without human trial data.

Despite their promising potential, several translational barriers must be acknowledged before these emerging strategies can be integrated into clinical practice. For gene therapy, stem cell-based approaches, and exosome-based treatments, concerns include off-target effects, tumorigenic risk, immunogenicity, and the lack of standardized manufacturing and quality control protocols. For nanomaterial-based and biomaterial-based strategies, long-term degradation profiles, local tissue toxicity, and batch-to-batch reproducibility remain unresolved. For antimicrobial and immunomodulatory agents, optimal dosing regimens and the risk of disrupting beneficial microbiota have not been systematically evaluated. Furthermore, most of these novel approaches are supported by preclinical animal data rather than human trials, and the high costs associated with their development and application may limit widespread accessibility. Regulatory pathways for these emerging therapies are also not well-defined, posing additional hurdles for clinical translation. Therefore, while the therapeutic potential is considerable, careful evaluation of safety, feasibility, and cost-effectiveness is essential before any of these strategies can be recommended for routine periodontal care in diabetic patients.

## 5 The future perspectives of diabetes mellitus on periodontal tissue defects

With the global rise in diabetes prevalence and the high incidence of diabetes-related periodontitis, current therapies still face challenges like incomplete blocking of the AGEs-RAGE axis, limited periodontal tissue regeneration in hyperglycemic environments, and insufficient precision in cross-system intervention. Future research should focus on building a “precision targeting-regenerative repair-clinical translation” chain through the following directions.

### 5.1 Precision targeting of core pathological pathways

The key lies in regulating key molecular targets. For the AGEs-RAGE pathway (the initiator of periodontal destruction), develop high-affinity RAGE antagonists that specifically block AGEs-RAGE binding, and optimize their pharmacokinetics (such as extending half-life via PEGylation) based on preclinical results showing reduced alveolar bone resorption in diabetic mice. For the AGEs-RAGE pathway, on the basis of developing high-affinity RAGE antagonists, local sustained-release formulations of advanced glycation end products (AGEs) formation inhibitors (e.g., pyridoxamine) and STAB1 receptor agonists can be combined to intervene in AGEs accumulation through three links: “inhibiting formation—blocking binding—promoting clearance”. This strategy is suitable for people with diabetes-related periodontitis who have a long-term glycated hemoglobin (HbA1c) level > 7.5% or a disease duration > 5 years.

For the ROS/MAPKs/NF-*κ*B inflammatory cascade activated by hyperglycemia, target key nodes like the NLRP3 inflammasome—use selective NLRP3 inhibitors (proven to lower pro-inflammatory cytokines in diabetic rats' periodontal tissues) and verify their clinical efficacy and long-term safety (134). Meanwhile, address abnormal non-coding RNA expression in diabetic periodontitis by developing nucleic acid-based drugs to regulate periodontal ligament stem cell (PDLSC) osteogenic differentiation at the transcriptional level, such as restoring high glucose-inhibited signaling pathways via targeted non-coding RNA upregulation (135, 136).

### 5.2 Optimization of periodontal tissue regenerative strategies

Integrate “microenvironment remodeling + seed cell enhancement” to solve the dual defects of pathological microenvironment and impaired seed cells. For microenvironment improvement, use hypoxia-adapted biomaterials (like hydrogels loaded with HIF-1*α* stabilizers) to promote angiogenesis—preclinical studies show such hydrogels can significantly increase vascular density in diabetic rats' periodontal defects in a short time (133, 137). For seed cell enhancement, apply exosome-based therapy: use non-diabetic bone marrow mesenchymal stem cell (BMSC) exosomes to restore diabetic BMSC osteogenic ability, modify exosomes to overexpress osteogenic molecules, and explore “exosome-biomaterial” composites (such as exosome-loaded electrospun membranes) for sustained release. Additionally, use 3D bioprinting to construct personalized scaffolds according to individual periodontal defect size and shape, load them with matrix degradation inhibitors and collagen synthesis promoters, and achieve “anti-degradation + pro-regeneration” effects.

### 5.3 Promotion of cross-system collaborative intervention

Establish a multi-disciplinary platform (including stomatology, endocrinology, and nutrition) and develop integrated intervention protocols based on diabetic people' glycemic control levels and periodontal conditions. On one hand, explore dual-effect drugs: encapsulate anti-diabetic drugs in periodontal local microspheres to enhance local concentration and avoid systemic side effects—clinical studies show local application can reduce periodontal probing depth in people diabetic periodontitis (138). On the other hand, build a “glycemic-periodontal” joint monitoring system: detect pro-inflammatory factors and advanced glycation end products in gingival crevicular fluid (which reflect efficacy earlier than clinical indicators like probing depth), combine with continuous glucose monitoring, and dynamically adjust treatment plans (such as modifying hypoglycemic drug doses or periodontal maintenance frequency) to break the “hyperglycemia-periodontal destruction” vicious cycle.

### 5.4 Acceleration of clinical translation

Establish a “preclinical validation-human trial-long-term follow-up” chain. First, use pathological animal models (such as db/db mice combined with ligature-induced periodontitis) to verify new therapies' efficacy and safety in periodontal tissue repair and metabolic regulation. Second, design strict phase I/II clinical trials (defining ranges for glycated hemoglobin and periodontal defect severity) to evaluate short-term efficacy (e.g., 3-month probing depth reduction) and long-term safety (e.g., 12-month adverse reaction rate). Finally, conduct follow-up studies lasting 5 years or more to track long-term effects on periodontal stability and glycemic control, build a real-world research database, and provide evidence for clinical guidelines and personalized treatment.

### 5.5 Artificial intelligence and remote monitoring in diabetic periodontitis management

Artificial intelligence and remote monitoring are emerging as transformative tools for diabetic periodontitis management. Machine learning models integrating continuous glucose monitoring data can predict glycemic fluctuations and guide proactive insulin adjustment, while deep learning algorithms applied to intraoral images enable early detection of alveolar bone loss and periodontal pocket progression. Unifying glycemic prediction with periodontal risk assessment into a digital platform allows real-time bidirectional monitoring of the diabetes-periodontitis axis. Remote monitoring systems have shown feasibility in maintaining periodontal stability between visits. Challenges include data standardization, algorithm generalizability, patient adherence, and regulatory approval. Future research should validate these digital tools in large-scale prospective cohorts.

## 6 Limitations

Several limitations of this review should be acknowledged. First, as a narrative rather than systematic review, this work may be subject to selection bias in literature inclusion, and the evidence synthesis is qualitative rather than quantitative. Second, the heterogeneity of diabetes models — including differences between type 1 and type 2 diabetes, variations in disease duration and glycemic control levels across studies — complicates direct comparisons and may limit the generalizability of our findings. Third, a substantial portion of the mechanistic evidence discussed herein is derived from *in vitro* high-glucose culture systems and animal models; translation of these findings to human diabetic periodontitis requires caution, as the complexity of the human hyperglycemic microenvironment cannot be fully recapitulated in experimental settings. Fourth, many of the emerging therapeutic strategies highlighted in this review are supported by preclinical data rather than robust clinical trials, and the possibility of publication bias — favoring positive results — should be considered when interpreting these findings. Finally, the high costs, regulatory hurdles, and long-term safety concerns associated with biomaterials, biologics, and gene-based approaches represent major barriers to clinical translation that have not yet been adequately addressed in the literature. Despite these limitations, this review provides a structured framework for understanding the pathogenic mechanisms and therapeutic landscape of diabetic periodontitis, while identifying key evidence gaps that warrant future investigation.

## 7 Conclusion

In summary, this review confirms that the hyperglycemic microenvironment drives periodontal tissue destruction through a multi-layered network involving immune cell dysfunction, osteoclast-osteoblast imbalance, impaired stem cell function, and cross-system mechanisms including the gut-periodontium axis and inflamm-aging. Among the therapeutic strategies reviewed, conventional approaches combining glycemic control with mechanical debridement remain the standard of care, supported by robust clinical evidence. Emerging therapies — including functionalized biomaterials, stem cell-derived products, and microbiome-targeted interventions — have demonstrated promising preclinical and early clinical potential, but their efficacy, safety, and cost-effectiveness require further validation in well-designed human trials. Future research should prioritize: (1) identifying reliable biomarkers to stratify patients for personalized therapy; (2) developing standardized protocols for cell- and exosome-based products; (3) conducting head-to-head comparisons between novel and conventional therapies; and (4) addressing translational barriers including regulatory pathways, long-term safety, and healthcare accessibility. By bridging mechanistic insights with clinical translation, this review aims to inform both researchers and clinicians about the current state of the field and the critical evidence needed to advance the management of diabetic periodontitis.

## Author statement

We declare that this manuscript is original, has not been published before and is not currently being considered for publication elsewhere.

We confirm that the manuscript has been read and approved by all named authors, and that the order of authors listed in the manuscript has been approved by all of us.

No persons or third-party services were involved in the research or manuscript preparation who are not listed as an author and have not been acknowledged.
